# Can a shoe-mounted IMU identify the effects of orthotics in ways comparable to gait laboratory measurements?

**DOI:** 10.1186/s13047-023-00654-8

**Published:** 2023-09-05

**Authors:** Max Lewin, Carina Price, Christopher Nester

**Affiliations:** 1 School of Health and Society, University of Salford, Manchester, UK; 2 Scholl’s Wellness Company, Hull, UK

**Keywords:** Orthotics, Footwear, Wearables, Sensitivity, Validation

## Abstract

**Background:**

Footwear and orthotic research has traditionally been conducted within laboratories. With increasing prevalence of wearable sensors for foot and ankle biomechanics measurement, transitioning experiments into the real-world is realistic. However wearable systems must effectively detect the direction and magnitude of response to interventions to be considered for future usage.

**Methods:**

RunScribe IMU was used simultaneously with motion capture, accelerometers, and force plates during straight-line walking. Three orthotics (A, B, C) were used to change lower limb biomechanics from a control (SHOE) including: Ground reaction force (GRF) loading rate (A), pronation excursion (A and B), maximum pronation velocity (A and B), and impact shock (C) to test whether RunScribe detected effects consistent with laboratory measurements. Sensitivity was evaluated by assessing: 1. Significant differences (t-test) and effect sizes (Cohen’s *d*) between measurement systems for the same orthotic, 2. Statistical significance (t-test and ANOVA) and effect size (Cohen’s *d* & *f*) for orthotic effect across measurement systems 3. Direction of orthotic effect across measurement systems.

**Results:**

GRF loading rate (SHOE: *p* = 0.138 *d* = 0.403, A: *p* = 0.541 *d* = 0.165), impact shock (SHOE: *p* = 0.177 *d* = 0.405, C: *p* = 0.668 *d* = 0.132), pronation excursion (A: *p* = 0.623 *d* = 0.10, B: *p* = 0.986 *d* = 0.00) did not significantly differ between measurement systems with low effect size. Significant differences and high effect sizes existed between systems in the control condition for pronation excursion (*p* = 0.005 *d* = 0.68), and all conditions for pronation velocity (SHOE: *p* < 0.001 *d* = 1.24, A: *p* = 0.001 *p* = 1.21, B: *p* = 0.050 *d* = 0.64).

RunScribe (RS) and Laboratory (LM) recorded the same significant effect of orthotic but inconsistent effect sizes for GRF loading rate (LM:* p* = 0.020 *d* = 0.54, RS: *p* = 0.042 *d* = 0.27), pronation excursion (LM: *p* < 0.001 *f* = 0.31, RS: *p* = 0.042 *f* = 0.15), and non-significant effect of orthotic for impact shock (LM: *p* = 0.182 *d* = 0.08, RS: *p* = 0.457 *d* = 0.24). Statistical significance was different between systems for effect of orthotic on pronation velocity (LM: *p* = 0.010 *f* = 0.18, RS: *p* = 0.093 *f* = 0.25).

RunScribe and Laboratory agreed on the direction of change of the biomechanics variables for 69% (GRF loading rate), 40%—70% (pronation excursion), 47%—65% (pronation velocity), and 58% (impact shock) of participants.

**Conclusion:**

The RunScribe shows sensitivity to orthotic effect consistent with the laboratory at the group level for GRF loading rate, pronation excursion, and impact shock during walking. There were however large discrepancies between measurements in individuals. Application of the RunScribe for group analysis may be appropriate, however implementation of RunScribe for individual assessment and those including pronation may lead to erroneous interpretation.

## Background

Orthotic and footwear testing requires the measurement of relevant variables to identify the changes in foot and lower limb biomechanics that are induced due to changes in design. Changes in foot pronation due to footwear and orthotic use have been widely investigated [1–3] due to its apparent association with lower limb injury [4]. Specifically, changes in the velocity and peak or range of foot pronation due to foot orthoses have been quantified [3, 5]. Additionally, changes in ground reaction force (GRF) loading rate have been used to quantify shock attenuation properties of orthotic devices [6, 7] and differing footwear midsole materials [8]. Impact forces and shock have also been used to assess differences in footwear (barefoot vs. traditional running shoes, [9]) and orthotic designs [6, 7, 10]. However, the above studies are laboratory-based experiments during straight line walking or running.

Wearable technology for the measurement of footwear and orthotic effects is not uncommon [11], examples include plantar pressure assessment [12, 13] and tibial accelerometers [14–17]. However, these are still utilised within a structured laboratory setting. Laboratories can be inaccessible due to high cost and skill requirements, whereas wearable technologies offer portability, low cost, reduced participant burden, and ease of application. However, this potential benefit can only be realised if wearables are able to output relevant variables with sensitivity in magnitude or direction to different footwear or orthotic designs.

The RunScribe is a commercially available IMU targeted towards use within runners to provide insight into running biomechanics. The units are affixed to the laces of the shoe of both feet and allow the participant to collect data on lower limb biomechanics outside of a traditional laboratory. Data are subsequently synchronised with a mobile phone app and processed using a pre-defined algorithm to provide data for each step during the collection period allowing users to view the peaks of each variable for each step or to view averages over the data collection period. The RunScribe is one of several wearable human gait technologies and provides temporospatial, GRF, acceleration, and foot pronation variables. These have all previously been used to evaluate the biomechanical effects of footwear and orthoses using traditional methods in laboratory settings [1, 3, 6, 7, 9, 10]. Previous validation studies show mixed agreement between RunScribe and comparable laboratory measures during running for pronation velocity (intraclass correlation coefficient (ICC) = 0.17—0.87) and pronation excursion (ICC = 0.40—0.57) [18], and impact shock (ICC = 0.38 – 0.54) compared to an accelerometer [16]. For walking, the RunScribe has been shown to have moderate agreement for pronation excursion, maximum pronation velocity, and GRF loading rate (ICC = 0.54—0.63) but low agreement for impact shock, braking shock, total shock and GRF (ICC = 0.18 – 0.35) compared with laboratory data [19]. RunScribe has also been used to identify differences in impact shock with neutral, minimalist, and maximalist footwear [16], and shown to be sensitive to changes in impact shock due to softer flooring surfaces and running speeds [20].

The RunScribe therefore offers potential promise in terms of validity of measures relevant to footwear and orthotic design when compared to laboratory measurements for some variables. Here we extend the understanding of its performance by investigating its ability to identify differences in biomechanical variables due to different orthotic designs to ensure its application to all variables for potential assessment is warranted.

## Aim

The aim of the study was to investigate the ability for the RunScribe inertial measurement unit (IMU) to detect the same orthotic effect as traditional laboratory measurements on/for ground reaction force loading rate, pronation excursion, maximum pronation velocity, and impact shock during walking.

## Methods

### Participants & protocol

Twenty participants (Male *n* = 8, Female *n* = 12) were recruited: age (33.6 ± 10.6 Years), height (1.71 ± 0.08 m), body mass (73.2 ± 11.9 kg) after ethical approval was granted by The University of Salford School of Health and Society ethics committee (application: 1391). Participants walked at self-selected speed in their own footwear as a control condition (SHOE), and three orthotic conditions A, B, and C (differing in shape, material, and proposed application) (Table 1), completing 5 walks producing 10 footsteps per foot in each orthotic condition (20 total footsteps).

**Table 1:** Orthotic conditions and design features

Orthotic Condition	Design Features	Photo (Right Foot)	Theoretical orthotic effect	Laboratory variables	RunScribe variables
A	- Medial arch support - Cushioned foam - Full length orthotic		Orthotic A reduces GRF loading rate compared to control condition. Orthotic A reduces pronation velocity and excursion compared to control condition	GRF loading Rate (N/s)	GRF loading rate (N/Kg/s)
B	- High medial arch support - Lateral cut out to forefoot - Deep heel cup - ¾ length orthotic		Reduces foot pronation excursion and maximum pronation velocity compared to the control condition and Orthotic A	Pronation Excursion (°) and maximum pronation velocity (°/sec) between initial foot contact to maximum foot pronation	Pronation Excursion (°) and maximum pronation velocity (°/sec) between initial foot contact to maximum foot pronation
C	- Gel Material - Heel only orthotic		Orthotic C reduces impact shock compared to the control condition	Peak positive vertical acceleration (Impact Shock (g))	Impact Shock (g)

### Laboratory measurements

**Fig. 1:**
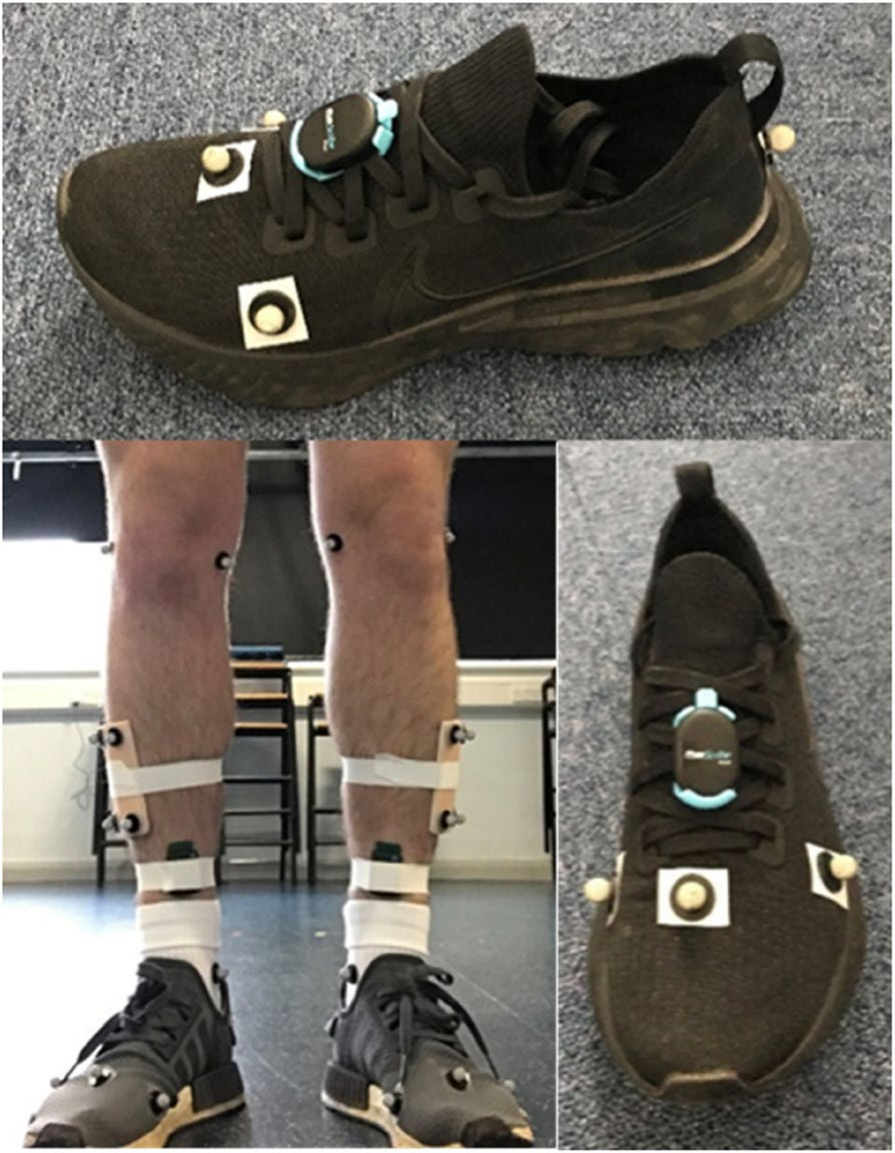
Marker and equipment placement on lower limbs and participant footwear

Participants walked across two 400 × 600 mm instrumented force plates (AMTI BP400600, Massachusetts, USA) operating at 1000 Hz with thirteen Qualisys Oqus cameras (Gothenburg, Sweden, 100 Hz) used for 3D motion capture. Markers were placed bilaterally on the: medial knee, lateral knee, medial malleolus, lateral malleolus, heel, metatarsal head (MH) 1, MH2, MH5, with the addition of a four marker cluster on the outer shank (Fig. 1) to enable the use of calibrated anatomical systems technique (CAST) [21]. All metatarsal head markers were placed on the shoe over the top of the anatomical landmark. As the RunScribe is a shoe mounted system, the markers were placed on the shoe to allow for the 3D motion to be tracked in the same way across both systems.

A Delsys Trigno Avanti (14 g) (Natick, Massachusetts, USA) sampling at 135 Hz was affixed bilaterally to the shank of participants approximately 10 cm above the medial malleolus. The attachment site was palpated to ensure the accelerometer was placed over the bony area of the shank avoiding areas of soft tissue. The accelerometer was positioned with the z-axis aligned to the axis of the tibia and fixed using double sided tape and subsequently wrapped using medical tape to reduce oscillation associated with foot contact. The Delsys accelerometer was started manually before each walk allowing it to operate simultaneously to the other laboratory measures.

### RunScribe measurement

The RunScribe IMU (Scribe Labs, Moss Beach, California, USA) sampling at 500 Hz was fitted to the laces of the footwear on both limbs as per the manufacturer instructions (Fig. 1). The RunScribe was started manually prior to the commencement of each walk allowing it to operate simultaneously with the force plates (force), Qualisys cameras (motion), and Delsys accelerometer (Shock).

### Data processing and synchronisation

Data from the RunScribe was processed from the RunScribe system using the mobile application and the pre-defined data processing algorithm available when using the IMU sensors. This processing method extracted a maximum value for each variable per step. The number of steps before the force plates was identified using data from the 3D motion capture system, this allowed for identification of the data from the RunScribe that corresponded to valid trials with contact with the force plates and were extracted for further analysis. Variables from the RunScribe for further analysis were GRF loading rate (N/Kg/s), pronation excursion (°), maximum pronation velocity (°/sec), and impact shock (g).

The same synchronisation process was followed for data from the Delsys accelerometer, where peak positive axial acceleration (along the tibia) was extracted to correspond to impact shock from the RunScribe IMU. Data from the force plates and 3D motion cameras were processed using Visual 3D (C-Motion, Maryland, USA). A Butterworth low pass filter was used on marker trajectories (10 Hz) and force data (20 Hz).

The shank was modelled with the proximal end defined using medial and lateral knee markers, and the distal with medial and lateral malleolus markers. The foot was modelled as a single segment with the proximal end defined using the malleolus markers and MH1 and MH5 defining the distal joint. These were projected to the floor to remove the offset through definition of a virtual foot and then the heel and three metatarsal markers were used for tracking. Pronation was defined as the rotation of the foot with respect to the shank in the frontal plane. Pronation excursion was defined as the range of pronation from initial contact to maximum pronation, and maximum pronation velocity was the maximum instantaneous velocity of joint rotation during this period. GRF loading rate was extracted from the force plate data.

Data was extracted from the left and right limbs from all systems, which was then averaged across both limbs to create a single participant average for comparison of the measures from the RunScribe and laboratory systems.

### Theoretical orthotic effect

Each orthosis was assessed for design features which may affect foot pronation, GRF or acceleration data in different ways (Table 1). A theoretical effect for each of the foot pronation, GRF loading rate, and impact shock variables was established based on the identified orthotic design features. This theoretical effect was tested using data from both the wearable and laboratory measures. Thereafter, a comparison of the outcome was made to answer the following question for each of the included variables.

Is a change in GRF loading rate, pronation excursion, pronation velocity, and impact shock in response to orthotic use detected by both measurement systems?

### Statistics

SPSS statistics 26 (IBM, New York, USA) was used to test differences between measurement systems (measure) and between orthotic conditions (orthotic). Data was checked for normality using a Shapiro–wilk test. Pronation excursion data was normally distributed without significant outliers. GRF loading rate data contained four individuals with outlying data, maximum pronation velocity data three outlying data points, and impact shock a single outlier. These data points were removed from further analysis therefore participant numbers for each variable were: GRF loading rate *n* = 16, pronation excursion *n* = 20, maximum pronation velocity *n* = 17, and impact shock *n* = 19.

To determine the ability for the RunScribe to accurately measure biomechanics variables, paired t-tests were used to compare variables across systems for the same condition. To determine the ability for the RunScribe to replicate results of orthotic testing from the laboratory system, paired t-tests were used to compare the control condition and test orthotic (GRF loading rate and impact shock). A repeated measures ANOVA was used for the same purpose when comparisons were required between the control condition and two orthotic conditions (pronation excursion and pronation velocity). Cohen’s D (*d*) effect size was calculated when a t-test was used and interpreted as: small (*d* = 0.20), medium (*d* = 0.50), and large (*d* = 0.80), Cohen’s F (*f*) effect size was calculated where an ANOVA was used and interpreted as: small (*f* = 0.10), medium (*f* = 0.25), and large (*f* = 0.40) [22].

To assess individual response to the orthotic and the ability for the measurement systems to detect this the difference between the orthotic conditions in each system was calculated. This was firstly used to identify the number of participants where the response to the orthotic occurred in the same direction for both systems. For further analysis, the absolute difference between this value from the RunScribe and the laboratory system was calculated to remove the influence of directional differences. This was used to assess both the magnitude of difference between the control and orthotic conditions, and the magnitude of difference between the two measurement systems.

### Evaluation criteria

RunScribe was said to be comparably sensitive to the orthosis effect vs. laboratory measurement approach when:

1. There was no statistically significant difference (*p* > 0.05) and low effect size (*d* = 0.2) indicated by the t-test between the measure given by the RS and the LM for the same orthotic condition.
2. There was matching statistical significance (ie. Both RS and LM show *p* ≤ 0.05 or *p* > 0.05) and effect size classification (low, medium, high) for orthotic effect.
3. The direction of the orthotic effect: Individual response to orthotics were assessed as control minus orthotic A, B, or C and orthotic A minus orthotic B, enabling the presentation of data for response to orthotic in both systems. This therefore enabled an analysis of the measurement systems to assess the ability for the RunScribe to detect the same response to orthotic use as the laboratory measurement system for all participants.

## Results

### Is an alteration in GRF loading rate in response to orthotic use detected by both measurement systems?

**Fig. 2:**
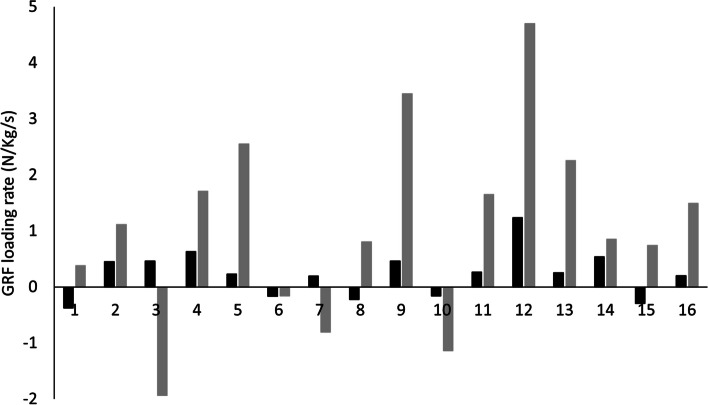
Difference in GRF loading rate between orthotic conditions (SHOE minus orthotic A) as measured by the RS (Black) and the LM (Grey)

The orthotic was successful in reducing GRF loading rate (*p* = 0.020 (LM) and *p* = 0.042 (RS)), with medium (*d* = 0.54) and small (*d* = 0.27) effect sizes respectively. There was no significant difference between the measurement systems (*p* ≥ 0.138) with small effect sizes for both SHOE (*d* = 0.17) and orthotic A (*d* = 0.40) (Table 2). Individual response to orthosis showed that LM and RS agreed on the direction of the change in GRF loading rate for 11/16 (69%) participants (Fig. 2). The average absolute difference between systems for response to orthotic was 1.42 N/Kg/s ranging from 0.01 N/Kg/s to 3.46 N/Kg/s when considering individual participant data.

**Table 2:** Ground Reaction Force loading rate research question data outcomes

GRF loading Rate (N/Kg/s)	Laboratory measurement (LM)	RunScribe (RS)	Mean difference (*P*-Value)	Effect Size (*d*)
SHOE	14.3 ± 2.2	14.0 ± 0.8	0.3 (0.541)	0.17
Orthotic A	13.2 ± 1.9	13.8 ± 0.9	0.6 (0.138)	0.40
Mean difference (*P*-Value)	1.1 (0.020)	0.2 (0.042)		
Effect Size (*d*)	0.54	0.27		

Data presented as Mean ± SD

Significance level set at *p* ≤ 0.05

### Is a change in pronation excursion and pronation velocity in response to orthotic use detected by both measurement systems?

There was significant main effect for orthotic (*p* < 0.001 (LM) and *p* = 0.042 (RS)) with medium (0.31) and small (0.15) effect sizes respectively. Both systems detected a significant reduction in pronation excursion when wearing orthotic B compared to orthotic A, however the RunScribe detected a significant reduction in pronation when wearing SHOE compared to both orthotic conditions whereas the LM did not. Significant difference (*p* = 0.005) was seen between systems for SHOE with a large effect size (*d* = 0.68), but no significant difference between the measurement systems for the orthotic conditions (*p* ≥ 0.623) and small effect sizes (*d* ≤ 0.10) (Table 3).

**Table 3:** Pronation excursion research question data outcomes

Pronation excursion (°)	Laboratory measurement (LM)	RunScribe (RS)	Mean difference (*P*-Value)	Effect Size (*d*)
SHOE	10.1 ± 1.9	8.1 ± 3.8 *^a,b^	2.0 (0.005)	0.68
Orthotic A	9.4 ± 2.4*^a^	9.4 ± 4.5*^a,c^	0.0 (0.986)	0.00
Orthotic B	8.5 ± 2.2*^a^	8.2 ± 3.9*^b,c^	0.3 (0.623)	0.10
ANOVA *P*-Value	< 0.001	0.042		
Effect Size (*f*)	0.31	0.15		

Data presented as Mean ± SD

Significance level set at *p* ≤ 0.05

^*^^a,b,c^ Identifies significant difference between conditions

**Fig. 3:**
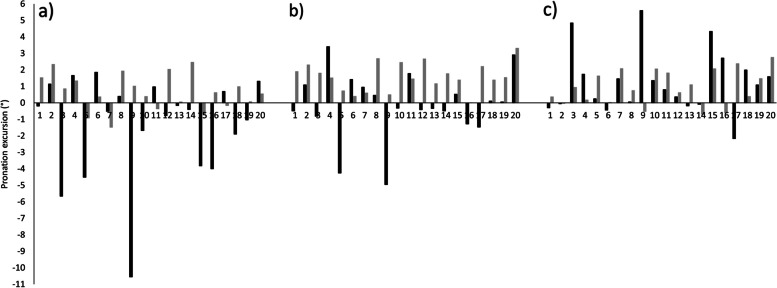
Difference in Pronation Excursion between orthotic conditions as measured by the RS (Black) and the LM (Grey). **a**) SHOE minus Orthotic A, **b**) SHOE minus Orthotic B, **c**) Orthotic A minus Orthotic B

The direction of the response to orthotic A compared to the control condition was the same between RS and LM for 8/20 (40%) participants (Fig. 3a) the average absolute difference between systems for response to orthotic was 2.6° ranging from 0.2° to 11.6°, only 10/20 (50%) of participants saw agreement between the two systems for direction of response to orthotic B (Fig. 3b) with the average absolute difference between systems for response to orthotic being 2.1° ranging from 0.3° to 5.5°. Comparing the orthotic conditions, 14/20 (70%) participants showed agreement between systems for response to wearing orthotic B compared to orthotic A (Fig. 3c), the average absolute difference between systems for response to orthotic was 1.6° ranging from 0.0° to 6.1°.

A significant main effect was seen between orthotic conditions on maximum pronation velocity when measured by the LM (*p* = 0.010) (*f* = 0.18 (small)), and significant difference between orthotic A and B (*p* = 0.048). This was not however identified by RS (*p* = 0.093) (*f* = 0.25 (medium)). There were significant differences between the LM and the RS measures of maximum pronation velocity across all test conditions (*p* ≤ 0.05) with large effect sizes (*d* ≥ 0.64). The RunScribe consistently recorded greater pronation velocities than the LM (mean differences 25.8—60.9°/sec) (Table 4).

**Table 4:** Maximum Pronation Velocity research question outcomes

Maximum pronation velocity (°/sec)	Laboratory Measurement (LM)	RunScribe (RS)	Mean Difference (*P*-Value)	Effect Size (*d*)
SHOE	181.2 ± 31.5	242.0 ± 61.7	-60.8 (< 0.001)	1.24
Orthotic A	196.7 ± 33.2^a^	222.4 ± 46.5	-25.7 (0.050)	0.64
Orthotic B	180.3 ± 29.1^a^	224.5 ± 41.1	-44.2 (0.001)	1.24
ANOVA *P*-Value	0.010	0.093		
Effect Size (*f*)	0.18	0.25		

Data presented as Mean ± SD

Significance level set at *p* ≤ 0.05

^a^ Signifies significant difference between conditions

**Fig. 4:**
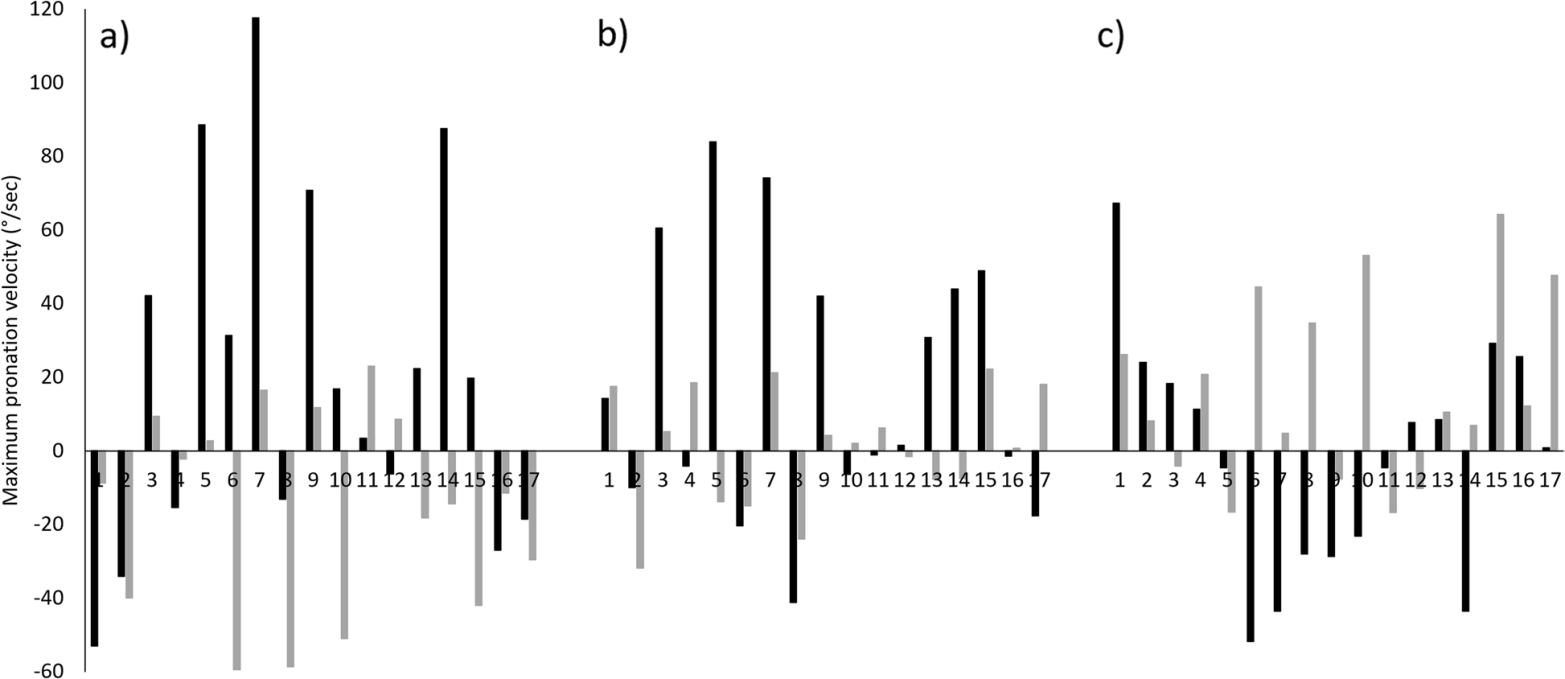
Difference in Maximum Pronation Velocity between orthotic conditions as measured by the RS (Black) and the LM (Grey). **a**) SHOE minus Orthotic A, **b**) SHOE minus Orthotic B, **c**) Orthotic A minus Orthotic B

The measurement systems agreed on the direction of change in pronation velocity when wearing orthotic A for 11/17 (65%) participants (Fig. 4a) the average absolute difference between systems for response to orthotic was 47.7°/sec ranging from 5.9°/sec to 101.9°/sec, correct direction of change was identified in 8/17 (47%) participants for orthotic B compared to the control (Fig. 4b) the average absolute difference between systems for response to orthotic was 28.7°/sec ranging from 2.1°/sec to 97.8°/sec. The change in pronation velocity due to the orthotics occurred in the same direction for RS and the LM in 10/17 (59%) participants when comparing orthotic A and orthotic B (Fig. 4c) the average absolute difference between systems for response to orthotic was 34.3°/sec ranging from 2.1°/sec to 96.3°/sec.

### Is a change in impact shock in response to orthotic use detected by both measurement systems?

There was no significant difference between SHOE and orthotic C in the RS or LM systems (*p* ≥ 0.18) with small effect size (*d* ≤ 0.24) (Table 5). There was no significant difference between the two measurement systems (*p* ≥ 0.177) and small effect sizes when comparing systems for the orthotic conditions (*d* ≤ 0.41).

**Table 5:** Impact shock research question outcomes

Impact shock (g)	Laboratory measurement (LM)	RunScribe (RS)	Mean Difference (*P*-Value)	Effect Size (*d*)
SHOE	1.4 ± 0.4	1.5 ± 0.4	0.1 (0.668)	0.13
Orthotic C	1.4 ± 0.4	1.6 ± 0.5	0.2 (0.177)	0.41
Mean Difference (*P*-Value)	0.0 (0.182)	0.1 (0.457)		
Effect Size (*d*)	0.08	0.24		

Data presented as Mean ± SD

Significance level set at *p* ≤ 0.05

**Fig. 5:**
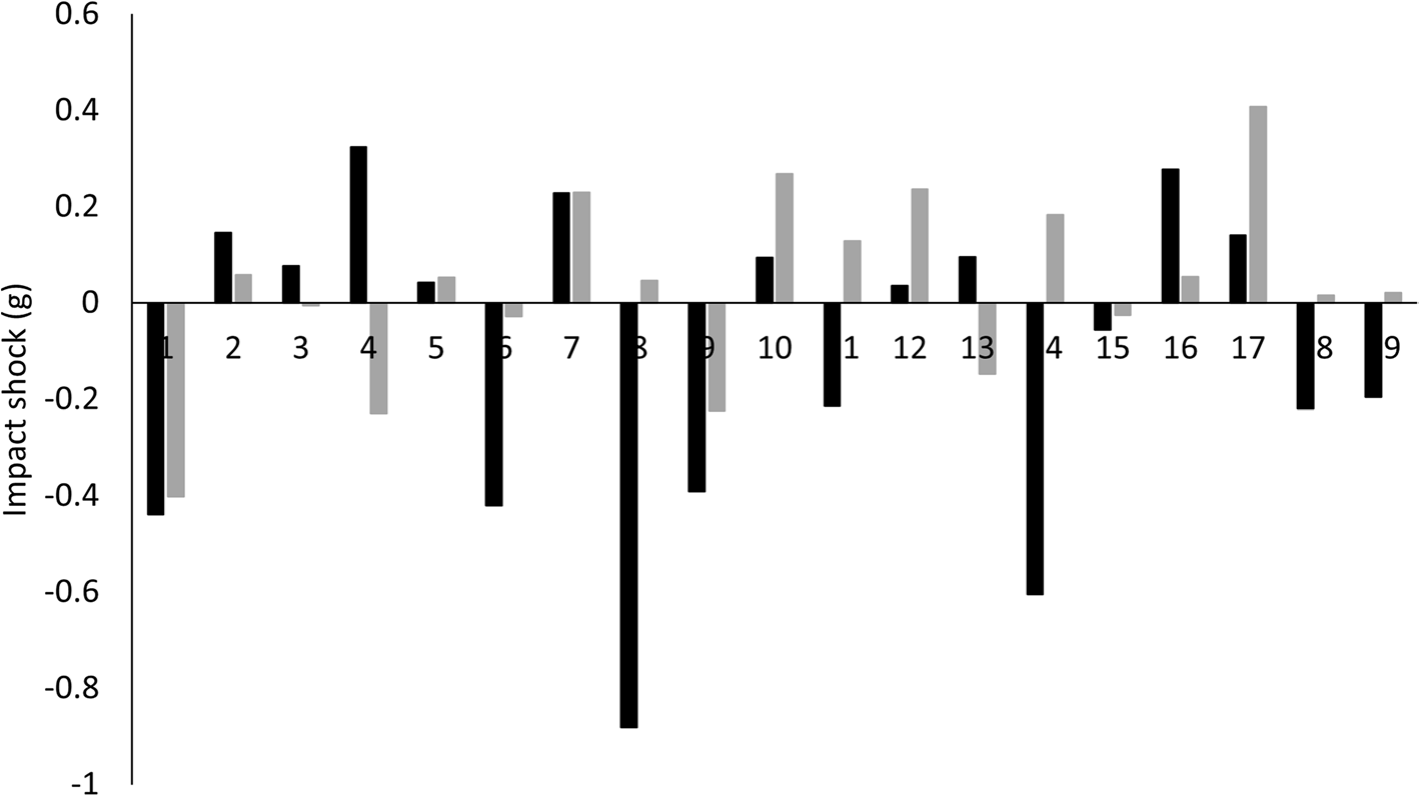
Difference in Impact shock between orthotic conditions (SHOE minus Orthotic C) as measured by the RS (Black) and the LM (Grey)

The measurement systems showed agreement for direction of change in response to orthotic C for 11/19 (58%) participants (Fig. 5) the average absolute difference between systems for response to orthotic was 0.26 g ranging from 0.00 g to 0.93 g.

## Discussion

Using the evaluation criteria to assess the RunScribe for each research question enabled the performance of the RunScribe for measurement of lower limb biomechanics to be compared to that.

of a traditional laboratory measurement system. Overall, the RS system showed capability as a tool to investigate orthotic and likely footwear effects for selected biomechanical variables. There were no significant differences between the RS and LM systems for GRF loading rate, impact shock, and for 2 out of 3 orthotic conditions for pronation excursion, therefore satisfying our first evaluation criteria. The second criteria was satisfied for GRF loading rate, impact shock, and pronation excursion by having the same statistical significance for orthotic effect in both systems. Effect sizes differed between systems, but were categorised at the same level (e.g. low, medium, or high [22]),. This criteria was however not satisfied for maximum pronation velocity. Our third criteria centre on the number of participants for whom the RS identified the same direction of orthotic effect as the laboratory measures. This ranged from 40—70% across the variables tested, and was over half of the sample with the exception of the effect of orthotic A on pronation excursion and orthotic B on maximum pronation velocity.

The practical utility of the RS for orthotic and footwear applications is supported by its ability to detect response to orthotic conditions across a sample population. In a standard group mean study design this would enable researchers, industry or clinicians to subsequently answer research questions. On the group level, the RS detects directional response in line with the traditional laboratory leading to the same conclusion for impact of orthotic use upon GRF loading rate, impact shock, and pronation excursion. However, when the data is examined on an individual level, the RS is less successful at detecting differences in response to orthotic, having lower utility within this area.

Results from the RS show a significant reduction in GRF loading rate due to the orthotic and small effect size (vs. medium effect size for the laboratory measurement). There was no significant difference between the RS and LM systems for measurement of the same orthotic condition, demonstrating comparable measurements from both systems. The RS also matched results from the laboratory measurement for GRF loading rate in 69% of participants. The agreement between systems was mixed at the individual level when considering the range of difference between systems when measuring orthotic effect (0.01 N/Kg/s to 3.46 N/Kg/s). Different footwear [23] and orthotic designs [7] have been effective in reducing GRF loading rate during running, as have different midsoles during drop landings [24] within which the results link reduced GRF loading rate to increased cushioning properties within footwear. Considering the current results, and the prevalent use of GRF loading rate within footwear and orthotic testing [6–8, 23, 24] the RS has a place testing such products on the group level due to replication of the statistical relationship between conditions being equivalent to those measured by the laboratory. Considering the use of the RS as a tool to measure an individual’s response to orthotics, the RS should be applied with caution in this scenario due to the mixed agreement with the laboratory measurement system.

Prior to testing, there was a strong potential use of the RS within research due to it’s potential to quantify pronation, the reduction of which is frequently an aim of footwear and orthotic interventions [1–3], custom made orthotics [25] and for treatment and prevention of lower limb pathologies [26]. There was no significant difference in pronation excursion between RS and LM for 2 out of 3 orthotic conditions and comparison of orthotic effect was also significant in both RS and LM measurement systems. The RS enables a difference in orthotic conditions to be detected in line with the laboratory measurements, therefore providing a suitable measure for group analysis of orthotic effect on pronation excursion. The RS correctly identified a maximum of 70% of individual relationships, recording a maximum difference between systems for response to orthotic of 11.6°. The low number of correct individual relationships here is related to differences between the RS and the laboratory measures in the control condition, whereas the results for the orthotic conditions are more comparable across the two systems. Within applications of the RS on an individual basis, current results showing a large number of incorrect decisions made regarding effect of orthotic could lead to an efficacious intervention to be deemed ineffective, or an ineffective intervention to be deemed as effective. Despite the pre-testing potential for RS to be implemented for the assessment of pronation, considering the current results and potential implications, application of the RS for assessment of pronation excursion at the individual level should therefore be considered with caution.

The RS was significantly different to the LM for maximum pronation velocity with large effect sizes in all comparisons across systems, and the RS measured the correct direction of change of maximum pronation velocity from orthotic use in 47%—65% of participants. Both systems defined the variable in the same period of stance and using frontal plane motion of the shoe/foot unit. The differing sample frequencies however would have influenced the velocity calculation, furthermore any error in excursion would result in potential error of velocity also. The magnitude of the individual differences also highlights the low agreement between the systems, with a maximum absolute difference of 101.9°/sec between the systems for the orthotic response. The average difference was 28.7°/sec to 47.7°/sec dependant on the conditions being compared, representing a large difference given the average group difference between conditions as measured by the laboratory was only a maximum of 16.4°/sec. This therefore means that the RS IMU is not suitable for measurement of maximum pronation velocity for either a standalone measurement of pronation, or a method for accurately detecting differences between orthotic conditions.

The RS provided a comparable measure of impact shock to the laboratory measurement for both conditions indicated by non-significant differences when comparing RS and LM systems, and small effect sizes. The differences in mounting location between the RS and the Delsys accelerometer (LM) within the method are expected to cause differences in magnitude of impact shock due to attenuation and the different axis of measurement [27]. However, there was no significant difference between SHOE and orthotic C in either of the measurement systems. The impact of orthotic usage was measured in the correct direction by the RS in 58% of participants, the average absolute difference between systems for orthotic effect was 0.26 g with a maximum difference of 0.93 g, representing a large difference between systems considering the small magnitude of impact shock measured and the absence of difference between conditions at the group level. As there is no difference between conditions when measured by the laboratory measurement, the RS shows the ability to provide the same results as the laboratory measurement system. The importance of a reliable measurement of impact shock within research is crucial due to widespread usage of shock absorbing insoles for prevention of lower limb injuries [26], field studies employing shock absorbing insoles have not tested the effectiveness of the interventions in vivo [28]. The RS would therefore enable a determination of insole effectiveness for shock absorption and prevention of subsequent injuries in this scenario. To fully understand the utility of the RS, it must be compared against the laboratory measurement when a difference is present between orthotic conditions. This will enable a conclusion to be drawn on whether the RS is sensitive to the changes in impact shock that an orthotic could create, enabling future implementation of the RS within orthotic testing.

Further influence on the difference between the laboratory and RS for all variables may relate to the footwear and fastening techniques used by each participant. Participants were responsible for lacing their own footwear, therefore lace pressure may not have been consistent across participants or conditions. As the RS is affixed to the laces, tight lacing would hold the RS firmly in place against the tongue of the footwear, loose lacing would lead to the RS not being rigidly fixed creating excessive movement in the RS alongside the shoes and associated markers. This may also impact the shock measured by the RS system due to increased oscillation and movement of the unit becoming uncoupled with the shoe. As this factor is dependent on the individual footwear fit and lacing pressure and will change from participant to participant, this may provide some explanation for some of the larger individual differences present within the results.

Effects of an orthotic on biomechanical outcomes are generally lesser in magnitude than that of a footwear intervention for example to control excessive pronation [25]. Furthermore footwear [9] is more effective than orthotics [7, 10, 29] for reduction of peak acceleration, and footwear also has larger effect on vertical loading rates [30] than orthotics [31]. As the differences associated with orthotics are relatively small, the questions posed of the RS within the current research provide a robust test of the RS measurements. The ability to detect the differences and similarities between orthotic interventions within the current results show promise for future application within footwear testing where differences between conditions are likely larger [25], allowing the measurement device to be less sensitive to measure the differences accurately. This would therefore enable both footwear and orthotic testing to be completed in the field allowing data to be captured and assessment of footwear and orthotic products to be completed within scenarios that are not accessible by traditional measurement methods. The results from the current study highlight that future application of RS should consider its capability to detect group differences in GRF loading rate, pronation excursion and impact shock between footwear conditions. RS is not recommended for experimental designs testing pronation velocity at the group level or testing individual patients. A limitation of the current study includes the effectiveness of orthotic C in reducing impact shock. Orthotic C displayed the same levels of impact shock as the control condition when measured by the laboratory measurement, it could therefore only be concluded that the RS is able to measure impact shock at a similar level to the traditional laboratory measurement. However further exploration of orthotic materials with different impact shock absorption properties can clarify the efficacy of RS compared to the lab across a range of shock values. Aims of the current investigation were surrounding the ability of the RS to accurately detect change, therefore use of an orthotic that has confirmed effectiveness in reducing impact shock over the control would enable an understanding of whether the RS is able to measure this difference, and satisfy the aims of the study. Further limitations include the low sampling frequency of the Delsys Trigno avanti accelerometer used as the laboratory measurement was undertaken through a mobile app. The low sampling frequency here may have led to aliasing error, missing the true peak of the positive acceleration meaning impact shock recorded by the laboratory system could be an underestimation.

The use of RS in the laboratory environment is an essential step in the research process and it has also let us explore its capability to address theoretical orthotic research questions. However constraining the assessment to a laboratory environment lacks the external validity of the real-world use of RS which is its main benefit. Similarly the results being captured over 20 steps as opposed over a days worth of walking on varied terrains and floorings needs to also be considered when applying RS to real world orthotic assessment.

## Conclusion

The RunScribe IMU delivers a solution to real-world gait measurement with suitable variables for the testing of footwear and orthotics. Assessing the RunScribe against the evaluation criteria set out within the methods showed promise for use at the group level to replicate statistical relationships seen using traditional laboratory measures for the effect of orthotics on GRF loading rate, pronation excursion, and impact shock. The RunScribe also provided a group average measurement of these variables that was not statistically significantly different to that of the lab alongside low effect sizes. There was however significant differences between systems for maximum pronation velocity and large effect sizes, the systems also did not show the same statistical effect of orthotic on maximum pronation velocity with varied effect sizes. Examination of individual differences show the RS to be unreliable at replicating results for individual response to orthotic usage, therefore implementation of the RS and interpretation of results should be done with caution. The benefits to using the RS system have been outlined in comparison to traditional biomechanics measurement using 3D motion capture, force plates, and accelerometers providing a real-world alternative to orthotic testing. However the results from the current investigation show that some usages of the RunScribe may lead to erroneous interpretation of orthotic effect.

## Data Availability

The datasets analysed during the current study are available from the corresponding author on reasonable request.

## Abbreviations

GRF: Ground Reaction Force
IMU: Inertial Measurement Unit
RS: RunScribe
LM: Laboratory measurement
